# Genomic profiling of the UFMylation family genes identifies UFSP2 as a potential tumour suppressor in colon cancer

**DOI:** 10.1002/ctm2.642

**Published:** 2021-12-19

**Authors:** Junzhi Zhou, Xiaohe Ma, Lu Xu, Qian Liang, Jian Mao, Jiang Liu, Miao Wang, Jiao Yuan, Yu‐sheng Cong

**Affiliations:** ^1^ Key Laboratory of Aging and Cancer Biology of Zhejiang Province School of Basic Medicine, Hangzhou Normal University 2318 Yu hang tang Rd Hangzhou Zhejiang 311121 China; ^2^ Department of Obstetrics and Gynecology University of Pennsylvania Philadelphia Pennsylvania USA

**Keywords:** SCNA, UFMylation, UFSP2

Dear Editor,

The ubiquitin and ubiquitin‐like protein modifications contribute to functional changes of substrate proteins and thus regulate a range of biological processes including cancer.^1^ Ubiquitin‐fold modifier 1 (UFM1) is a newly identified ubiquitin‐like modification with essential biological functions.^2^, ^3^ Like ubiquitination, UFM1 modification (UFMylation) is catalyzed by the dynamic E1–E2–E3 enzymatic reaction. Unlike ubiquitination which encompasses several E1s and different E2s (>50) and E3s (>600), only one of each enzyme, UBA5 (E1‐like), UFC1 (E2‐like), and UFL1 (E3‐like), has been identified for the UFMylation reaction.^2^, ^3^ The proteases UFM1‐specific cysteine proteases 1 (UFSP1) and 2 (UFSP2) execute the maturation of the UFM1 precursor and de‐UFMylation process.^2^, ^3^ In addition, the satellite components DDRGK1 and CDK5RAP3 are considered as key regulators of the UFMylation system.^3^, ^4^, ^5^ Recent studies suggest that UFMylation plays critical roles in diverse cellular processes.^3^ However, the roles of UFMylation in tumorigenesis have not been systematically explored. In this study, we comprehensively analysed the genomic alterations of these eight UFMylation family genes across the Cancer Genome Atlas (TCGA) data cohort. (Data S1). We performed GISTIC2.0 analysis and identified a total of 55 recurrent and focal somatic copy number alterations (SCNA) events in UFMylation family genes across the whole TCGA cohort with 33 cancer types (Figure 1A). Among the UFMylation genes, UFSP2 was frequently deleted in 14 cancer types. We calculated the frequencies of copy number gain or loss of UFMylation genes in each cancer type (Figure 1B). We found UFSP2 (31%), UFM1 (31%) and UFL1 (28%) showed the highest average frequency for copy number loss, whereas UFC1 (34%), UFSP1 (34%) and DDRGK1 (30%) had the highest average alteration frequency for copy number gain (Figure 1B). Similar SCNAs patterns of the UFMylation genes were observed by using the ICGC Data Portal (Figure S1 and Data S2). Interestingly, we found that the UFSP2 copy number was mainly heterozygous loss in tumours and cancer cell lines (Figure S2), and homozygous deletions were rarely detected, indicating a critical biological function and possible haploinsufficiency of the UFSP2 gene (Figure 1A and Data S3).

**FIGURE 1:**
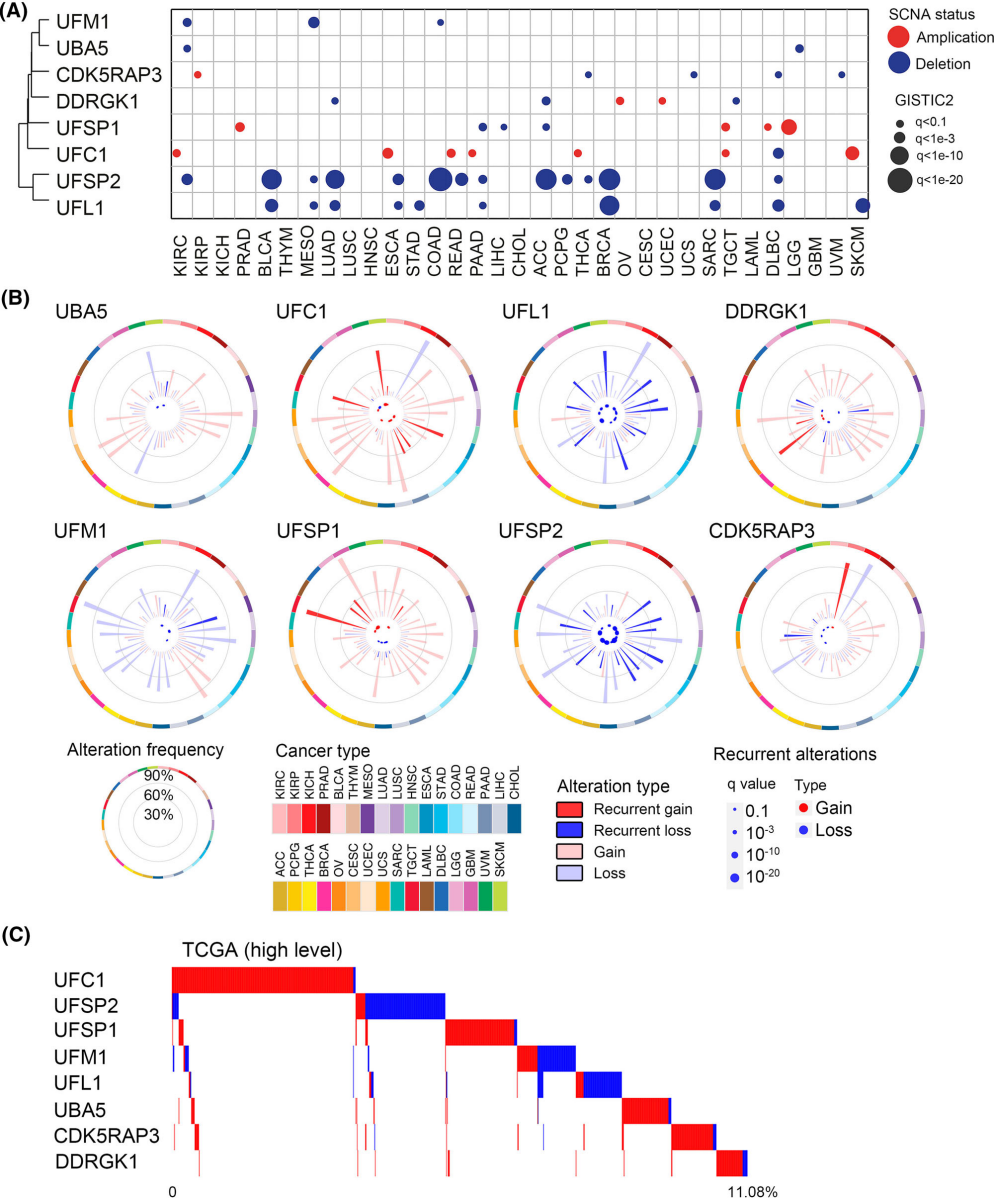
Somatic copy number alterations of UFMylation family genes in cancers. (A) Focal recurrently amplified and deleted UFMylation gene across 33 cancer types. The circle size is proportional to the significance level of GISTIC2 results. Red: amplification; blue: deletion. (B) Frequency of gain or loss of UFMylation genes in individual cancer types. Polar grid lines correspond to the frequency of 30%, 60%, and 90%, respectively. In the centre of each circle, the cancer types that harboured recurrent alterations of the given UFMylation gene are indicated by a colour‐coded bubble (red: gain; blue: loss). The size of the bubble plot represents the significance assessed by GISTIC2. The most outer layer indicates cancer types by colour. (C) Overview of amplifications and deletions in UFMylation genes across TCGA tumour samples (red: amplification; blue: deletion)

To further explore the SCNA patterns of UFMylation genes, we calculated the high‐level alteration of SCNAs and found that 11.08% of TCGA samples have high‐level copy number alterations in at least one of the eight genes. Noteworthy, the high‐level amplifications and deletions of UFMylation genes appeared to be occurred in a pattern of mutually exclusive (Figure 1C), suggesting UFMylation genes share common biological functions. However, we noticed that most of UFMylation genes exhibit low frequencies of somatic mutations (<5%) and transcript fusions (<.1%) (Figure S3 and Data S4–S7). Gene expression profiles analysed based on the RNA sequencing data from TCGA database (Data S8 and S9) indicate that UFMylation genes were ubiquitously expressed (Figure S4).

Although most UFMylation genes generally have low mutation frequencies in cancers, we found that UFSP2 is frequently mutated in colon adenocarcinoma and uterine corpus endometrial carcinoma. The high recurrent copy number loss and frequent somatic mutations of UFSP2 suggest that UFSP2 may function as a tumour suppressor. It has been previously demonstrated that knockdown UFSP2 promoted breast cancer cell growth and tumour formation, suggesting that UFSP2 is a tumour suppressor in breast cancer.^6^ In addition, we found that the levels of UFSP2 mRNA were significantly lower in 11 cancer types (Figure S5A). Using human tissue microarrays, we confirmed that the expression of UFSP2 was significantly reduced in cancer tissues (Figures 2A and S6). To further validate the potential tumour suppressor function of UFSP2 in colon cancer, we examined the effects of UFSP2 knockdown on cell growth of colon cancer cells HT29 and HCT116, and observed that knockdown UFSP2 expression significantly promoted growth rates of colon cancer cells and its anchorage‐independent cell growth. (Figure 2B–G). Significantly, knockdown UFSP2 expression significantly promoted the growth of xenograft tumours from UFSP2 depleted HT29 cells (Figure 2H–J). In addition, we observed that total UFMyaltion levels were increased in both UFSP2 depleted cells and xenograft tumours (Figure S7). These findings suggested that genomic alterations of UFSP2 were associated with the functional involvement of UFMylation in human colon cancer. Furthermore, our GSEA analysis indicates that loss of UFSP2 is mainly associated with the pathways in DNA replication, cell cycle, spliceosome, ribosome and mismatch repair (Figure S5C–D and Data S10). This is in line with previous reports that the UFMylation is critically involved in DNA damage, cell cycle, and ribosome protein modification.^3^, ^4^, ^7^, ^8^ We confirmed that knockdown UFSP2 increased the expression of some marker genes in DNA replication (PCNA and MCM2), cell cycle (CDK4 and CCND1), and ribosome protein (RPL26) (Figure S5E). It has been reported that MCM2, CDK4, and PCNA are interacted with UFM1 and are potential targets of the UFMylation.^7^ Thus, we speculate that UFSP2 may contribute to the tumorigenesis by modulating key regulators of cell cycle, DNA replication or protein biogenesis through Ufmylation modification. Together, our experimental findings revealed that knockdown UFSP2 expression significantly promotes the growth of colon cancer cells both in vitro and in vivo, suggesting that UFSP2 is a potential tumour suppressor in colon cancers. Given that UFMylation genes exhibit a high frequency of SCNA with a mutually exclusive pattern in common adult cancers, this suggests that involvement of UFMylation in cancer may be cell type or tissue specific. In line with this, it has been previously reported that CDK5RAP3 may function as a tumour suppressor^9^ in HNSCC and as an oncogene in liver cancer.^10^

**FIGURE 2:**
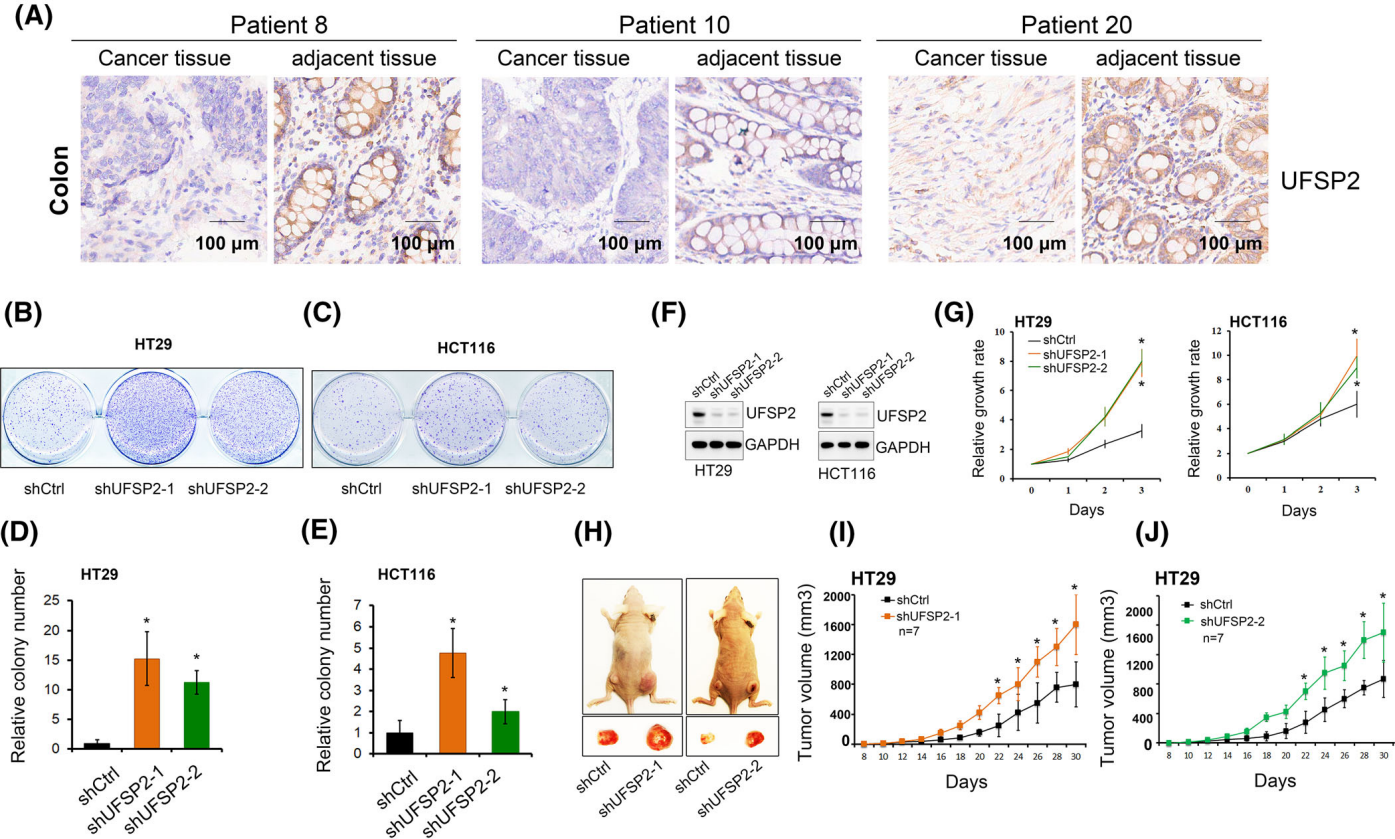
Identification of UFSP2 function as a potential tumour suppressor in colon cancer. (A) Representative sections of immunohistochemical staining of UFSP2 in colon cancer patients in tissue array. Soft agar assays of HT29 (B) and HCT116 (C) expressing control and shUFSP2‐RNAs. Quantification of the relative number of colonies from the soft agar assays on HT29 (D) and HCT116 (E). (F) UFSP2 was knocked down with two shRNAs in HT29 and HCT116 and knockdown efficiencies were confirmed by western blot. (G) Growth curves of colon cancer cell lines depleted UFSP2 by specific shRNAs. (H—J) In vivo xenograft tumour growth curve of HT29 expressing control and UFSP2‐shRNAs and representative pictures of tumours derived from mice. *t‐test *p*‐value < .05

In summary, we found that UFMylation family genes have a high frequency of SCNAs. Especially, UFSP2 is recurrently and focally deletion in a total of 14 human common cancers with the highest alteration score. We further demonstrated that depleted UFSP2 expression significantly promotes the growth of tumour cells both in vitro and in vivo, suggesting that UFSP2 may function as a tumour suppressor in colon cancers. Our integrated genomic analysis and functional studies provide insights in understanding this new post‐translational modifier in cancer and potential therapeutic opportunities.

## Supporting information



Supporting information
