# Supplementary material for: Genomic profiling of the UFMylation family genes identifies UFSP2 as a potential tumour suppressor in colon cancer
Source: Clin Transl Med. 2021 Dec 19;11(12):e642. doi: 10.1002/ctm2.642 (PMC8684770; doi:10.1002/ctm2.642)
Supplement: Supplementary file 1 — Supporting information [file CTM2-11-e642-s001.zip › Materials and methods.docx]

**Materials and methods**

**Recurrent focal SCNA estimation**

Recurrent SCNAs identified in each cancer type determined by GISTIC2.0 with the hg19 reference build were downloaded from Broad Institute GDAC Firehose (<https://gdac.broadinstitute.org/>). UFMylation family genes located within GISTIC peaks were considered SCNA-associated cancer drivers. Gene-level copy number values at cancer type-specific level and at pan-cancer level were downloaded from Broad Institute GDAC Firehose (<https://gdac.broadinstitute.org/>) and GDC (<https://gdc.cancer.gov/about-data/publications/pancanatlas>), respectively. CNA data of cancer cell lines were extracted from CCLE database.

**Validation of SCNAs of UFMylation genes in ICGC database**

Segmentation files of ICGC tumor samples were retrieved from ICGC Data Portal (https://dcc.icgc.org/releases/PCAWG/; retrieval date: Oct, 30, 2021). The Genomic Identification of Significant Targets in Cancer (GISTIC 2.0) algorithm (https://www.broadinstitute.org/cancer/cga/gistic) was used to identify significantly recurrent focal genomic regions that were gained or lost in a given tumor type using the segmentation files as input. GISTIC deconstructed copy number alterations into broad and focal events and applied a probabilistic framework to identify location and significance levels of SCNAs. For recurrent focal SCNA estimation, the significance levels (q values) were calculated by comparing the observed gains/losses at each locus to those obtained by randomly permuting the events along the genome. Amplifications and deletions were considered separately. Tumors which had more than 2,000 segments were excluded from our analysis. Default parameters of GISTIC were used with the confidence level set to 0.95 (by-conf). Focal events with q-value below 0.25 were considered as significantly recurrent.

**Gene expression analysis**

To evaluate the UFMylation family genes expression level across cancer transcriptomes,

matrix of gene-level FPKM (fragments per kilobase of transcript per million mapped reads) of tumor specimens across 33 cancer types from 27 primary sites were downloaded from the GDC Data Portal (<https://portal.gdc.cancer.gov/>) (retrieved date: 17 July 2019). If more than one sample existed for a participant, one single tumor sample was selected based on the following rules: (1) tumor sample type: primary (01) > recurrent (02) > metastatic (06); (2) order of sample portions: higher portion numbers were selected; and (3) order of plate: higher plate numbers were selected. R package “tSNE” was used to cluster all tumor samples across 33 TCGA cancer types on log2 (FPKM+1) values of UFMylation genes.

**DNA methylation analysis**

DNA methylation data at pan-cancer level generated by Illumina Infinium HM450 array (485,577 CpG site targeting probes) were downloaded from Genomic Data Commons (GDC) (<https://gdc.cancer.gov/about-data/publications/pancanatlas>) (retrieved date: 26 September 2018). For each UFMylation family gene, cancer-specific correlation of gene expression with the DNA methylation level were calculated for each probe in CpG sites. For each cancer type, we picked single probe which had the most negative average correlation coefficient to represent methylation levels of the nearest gene. Samples with a beta value > 0.3 in picked probes were marked as methylation-positive gene in each sample. We then plotted the fraction of samples positive for DNA methylation within each tissue type, tumors and adjacent normal tissues separately.

**Whole-exome sequencing data collection and processing**

We obtained TCGA pan-cancer somatic mutation data from GDC (<https://gdc.cancer.gov/about-data/publications/pancanatlas>). Mutation Annotation Format (MAF) profiles for 33 cancer type were downloaded from the TCGA MC3 project. The MC3 data were generated through seven independent mutation calling algorithms, including Pindel (INDEL), MuSE (SNV), Radia (SNV), VarScan2 (SNV/INDEL), MuTect (SNV), Indelocator (INDEL), and SomaticSniper (SNV). Variants from each caller were merged, quality control filtered, and stored in MAF file. If multiple samples existed for a participant in the MAF, one single pair of tumor/matched control sample was kept following the rules: (1) sample type: for tumor tissues, primary (01) > recurrent (02) >metastatic (06); for normal tissues, blood (10) > solid (11); (2) molecular type of analyte for analysis: prefer D analytes (native DNA) over G, W, or X (whole-genome amplified); (3) order of sample portions: higher portion numbers were selected; and (4) order of plate: higher plate numbers were selected. We excluded all mutations that were not tagged with PASS or WGA alone in all cancer types.

**Somatic mutation analysis**

Non-synonymous mutations mapped to UFMylation family genes were selected and filtered according to the functional annotation. In brief, mutations not affecting the sequence of the encoded protein (including mutations in UTR regions or UTR flanking regions, mutations in introns or non-coding RNAs, and synonymous mutations) were removed. Only mutations tagged with FILTER values of “PASS” or “WGA” were kept, leaving a final list of 414 mutations in the UFMylation family genes for downstream analysis. We searched all synonymous mutations in UFMylation genes in three Database rsynmicdb, IDSV and PrDSM. We collected INDEL in TCGA data from cbioportal. 60 indels found across 23 cancer types.

**Transcript fusion analysis**

Transcript fusions involving UFMylation family genes were downloaded from the TCGA fusion database (<https://tumorfusions.org/>) which was derived using PRADA. Each fusion was classified as one of the following tiers based on level of evidence: tier 1, tier 2, tier 3, and tier 4. Genomic distribution of break points in fusions events were visualized by circos.

**Biological pathway analysis**

In order to identify pathways associated UFSP2 copy number loss, we used linear regression models (limma) to reveal the associations between UFSP2 copy number and gene expression in each cancer type with recurrent UFSP2 copy number loss. A pre-ranked gene list was built based on t statistics for each cancer type. For gene-set enrichment analysis (GSEA), the pre-ranked gene lists were then run against gene sets derived from KEGG pathway database. The same analysis was also performed at pan cancer level. Pathways were ranked by the normalized enrichment scores (NES) with a significant correlation (q < 0.05 and FDR <0.25).

**Cell culture and reagents**

HT29 cells were maintained in DMEM (BI, Biological Industries) with 10% FBS (Hyclone). HCT116 cells were maintained in McCoy’s 5A medium (BI) with 10% FBS.

The antibodies used in this study included anti-GAPDH (HuaAn Biotechnology, M310-2) and anti-UFSP2 (Abcam, ab185965). Target sequences used for UFSP2-shRNAs were: AATAACTTGCAGGTCTTCAGC for UFSP2-1; TTTCATTAGGAGTAGCTAGC for UFSP2-2.

**shRNA knockdown and lentiviral transduction**

Lentiviral shRNAs targeting UFSP2 were constructed based on the pLKO.1 empty vector. Non-target shRNA was used as controls. Lentiviral vectors and packaging vectors (psPAX2 and pMD2.G) were transfected into 293T using the Lipofectamine 3000 (Invitrogen). The media was changed 8 hrs post transfection and the media containing lentivirus was collected 48 h later. Cancer cells were infected with lentivirus in the presence of 8 μg/ml polybrene (Sigma). Knockdown efficiency was detected 48 hrs after infection by western blot.

**Soft agar assay**

HT29 and HCT116 were seeded into 0.4% top agarose solution at density of 3×10^3^ and 2×10^3^, respectively, then carefully placed on top of the bottom agarose (0.8%) in the six-well plates. The plates were incubated at 37 °C with 5% CO2 until colonies were formed. Cell culture medium then was changed one to two times per week. After 3-4 weeks, cell colonies were stained using 0.1% crystal violet solution and relative colony intensity was quantitated by Image J image processing program.

**Mouse xenograft model in vivo**

Six-week-old BALB/c nude mice were used in the xenograft assays. HT29 cells were trypsinized and suspended in phosphate-buffered saline (PBS), then a total volume of 0.1 ml PBS containing HT29 cells (3×10^6^) were injected subcutaneously into the mouse flank. Four weeks after injection, mice bearing tumors were sacrificed by cervical dislocation for the assessment of tumor size examination. Tumor volumes were calculated by the formula V = (4/3) π×r^3^, where r is the radius of a tumor. All animal experiments were performed according to guidelines of Animal Care and Use Committee of Hangzhou Normal University.

**Tissue microarray and immunohistochemistry**

The tissue microarrays of human colon and kidney tumor samples were purchased from Shanghai Outdo Biotechnology. Immuno-histochemical staining was performed with primary antibodies against UFSP2 (Abcam, ab185965). The immunostaining was scored by pathologists in a blinded manner. A four-tier grading system (0, negative; 1, weak; 2, moderate; and 3, strong staining intensity) was used.

**Statistical analysis**

Statistical analysis was performed using R software. All results were expressed as mean ± SD and p < 0.05 were considered significant.

**Data availability**

The data that support the conclusions of this study are available from the corresponding author upon request.
