# Supplementary material for: Genomic profiling of the UFMylation family genes identifies UFSP2 as a potential tumour suppressor in colon cancer
Source: Clin Transl Med. 2021 Dec 19;11(12):e642. doi: 10.1002/ctm2.642 (PMC8684770; doi:10.1002/ctm2.642)
Supplement: Supplementary file 1 — Supporting information [file CTM2-11-e642-s001.zip › Supplementary figures.pdf]

Supplementary Figures

Supplementary Fig. S1. SCNAs of UFMylation family genes in ICGC

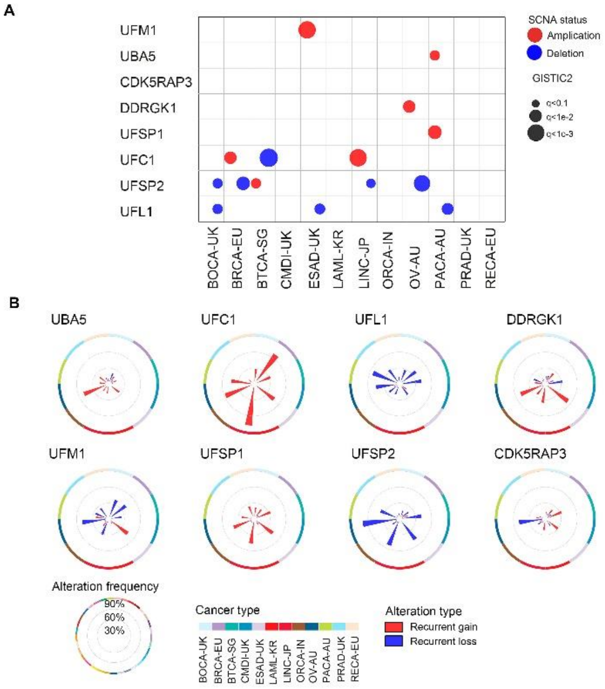

A. Focal recurrently amplified and deleted UFMylation gene across 12 cancer types. The circle size is proportional to the significance level of GISTIC2 results. Red: amplication; blue: deletion. B. Frequency of gain or loss of UFMylation genes in individual cancer types. Polar grid lines correspond to frequency of 30%, 60%, and 90%, respectively. In the center of each circle, the cancer types that harbored recurrent alterations of the given UFMylation gene are indicated by a color-coded bubble (red: gain; blue: loss). The most outer layer indicates cancer types by color.

Supplementary Fig. S2. UFSP2 SCNA in multiple cancer cell lines

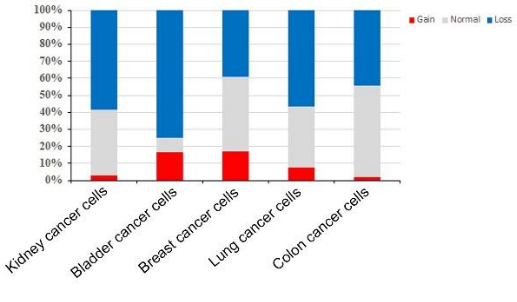

A. Focal recurrently amplified and deleted of UFSP2 gene based on CCLE database. Percentage stands for alterations of amplified, deleted, and normal SCNA of UFSP2 within the total cell lines. Red: Gain; blue: loss. Grey: Normal.

Supplementary Fig. S3. Somatic mutations and transcript fusions of UFMylation family genes in cancers

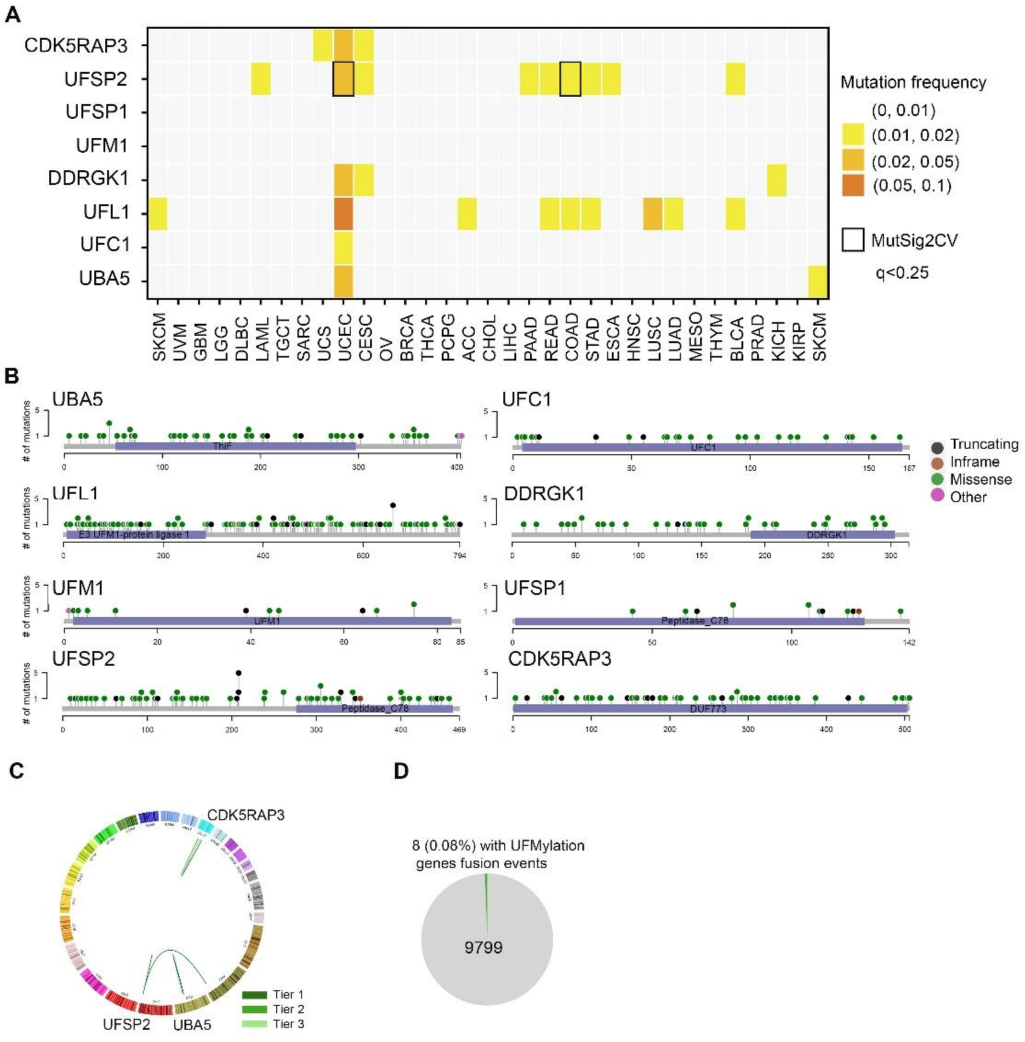

A. The mutation frequency heatmap of UFMylation genes in each cancer type. The intensity of yellow indicates mutation frequency, with higher mutation frequency in a cancer type indicated by darker color, and significantly mutated genes identified by MutSig2CV (q < 0.25) are highlighted in black boxes. B. The lollipop plots illustrate the distribution and categories of somatic mutations in each UFMylation genes. Mutation categories: black, truncating; orange, inframe; blue, missense. C. The fusions of UFMylation gene in TCGA samples are shown in Circos plots. The fused genes are illustrated as lines that connect two parental genes. The intensity of the line corresponds to the tier of the fusion event. D. Percentage of tumor specimens with UFMylation gene transcription fusion events across all cancer types.

Supplementary Fig. S4. Ubiquitous mRNA expression of UFMylation family genes in human cancers

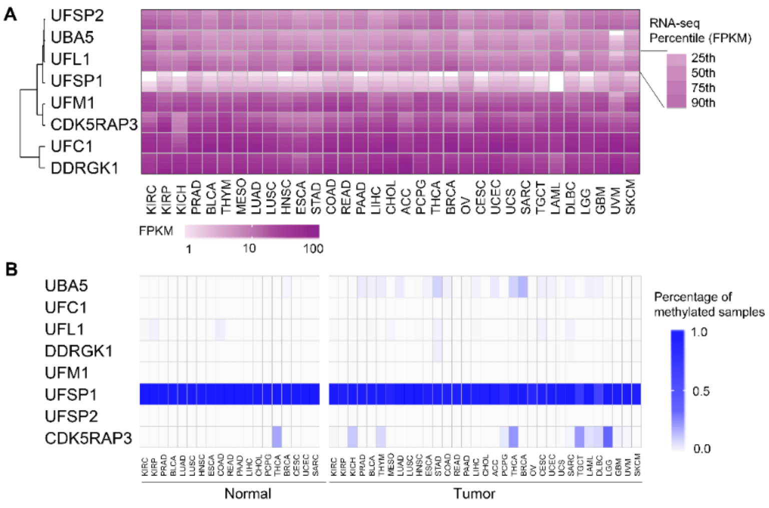

A. The heatmap shows the mRNA expression levels of UFMylation gene across cancers. The intensity of purple indicates the percentile (25th, 50th, 75th, and 90th) of the FPKM value of each UFMylation gene in a given cancer type. B. The heatmap shows the methylation of each UFMylation gene promoter. The intensity of blue indicates the percentage of methylated samples. *<0.05, **<0.01, and ***<0.001.

Supplementary Fig. S5. Biological pathways associated with UFSP2 SCNA in human cancers

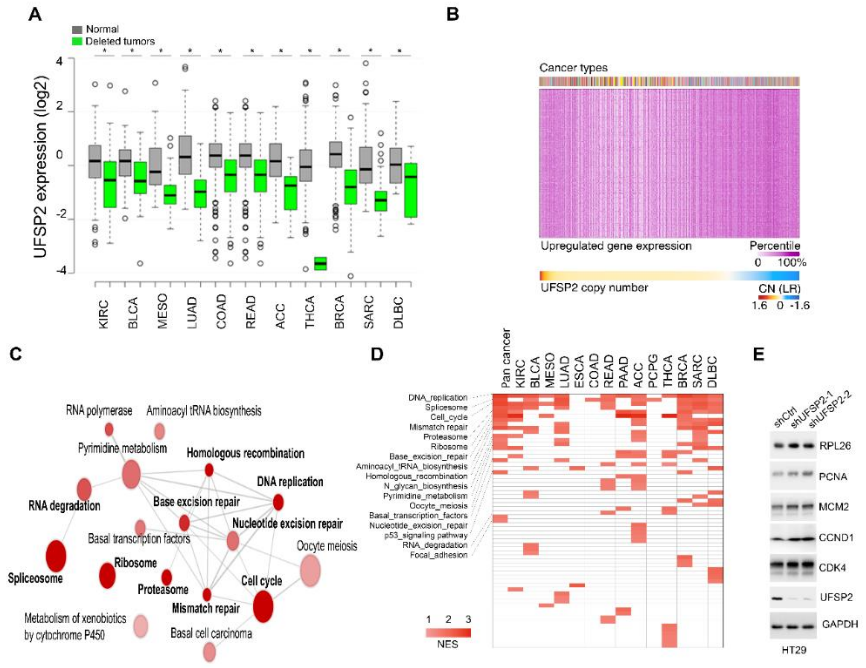

A. Decreased expression of UFSP2 mRNA in multiple cancer types. Gray: tumor tissues with normal UFSP2 copy number; blue: tumors with UFSP2 deletion. SCNA of UFSP2 in each tumor specimen was estimated by a GISTIC2.0 analysis. B. The heatmap of the genes that were significantly, positively co-expressed with UFSP2 loss in eleven cancer types. Each tumor is represented in a column and each gene in a row. The copy number of UFSP2 is presented as a bar graph under the heatmap. The genes were ranked from top to bottom in descending order of their correlation with UFSP2. C. The functional hubs of functional pathways associated with UFSP2 expression as analyzed by pre-ranked GSEA. D. The pathways represented for UFSP2 loss-associated genes at the pan-cancer and individual cancer levels, according to pre-ranked GSEA analysis. E. The expression of marker genes of cell cycle and DNA replication were examined by Western blot with indicated antibodies.

Supplementary Fig. S6. Reduced expression of UFSP2 in kidney cancers

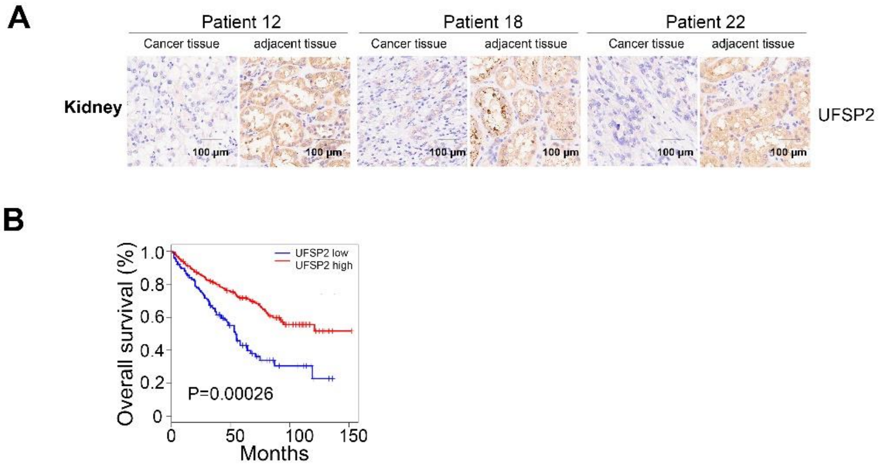

A. Representative sections of immunohistochemical staining of UFSP2 in KIRC patients in tissue array. B. The correlation between UFSP2 expression and overall survival rate through Kaplan-Meier analysis in patients with kidney renal clear cell. The overall survival rates of the high (top 50 percentile) and low (bottom 50 percentile) UFSP2 expressers are compared using the TCGA provisional datasets of KIRC (kidney renal clear cell carcinoma) (P=0.00026, n=261) for overall survival by the two-sided Mantel-Cox Log-Rank test.

Supplementary Fig. S7. Down-regulation of UFSP2 increased the global levels of UFMylation in cells and xenograft tumors

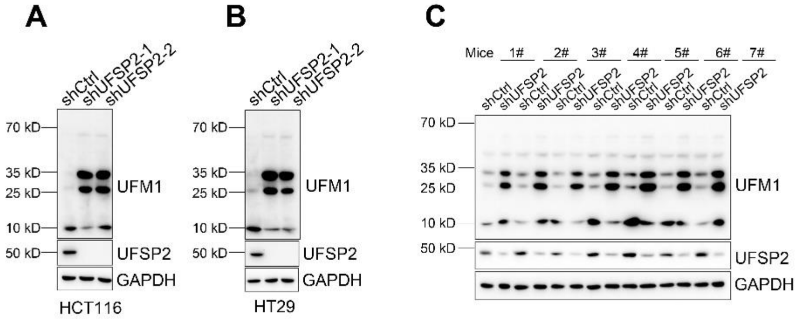

Western blot analysis of UFSP2 expression and the global levels of the UFMylation in colon cancer lines HCT116 (A), HT29 (B), and xenograft tumors (C).
